# What factors predict length of stay in a neonatal unit: a systematic review

**DOI:** 10.1136/bmjopen-2015-010466

**Published:** 2016-10-18

**Authors:** Sarah E Seaton, Lisa Barker, David Jenkins, Elizabeth S Draper, Keith R Abrams, Bradley N Manktelow

**Affiliations:** 1 Department of Health Sciences, University of Leicester, Leicester, UK; 2 Neonatal Unit, University Hospitals of Leicester NHS Trust, Leicester, UK

**Keywords:** NEONATOLOGY, EPIDEMIOLOGY

## Abstract

**Objective:**

In the UK, 1 in 10 babies require specialist neonatal care. This care can last from hours to months depending on the need of the baby. The increasing survival of very preterm babies has increased neonatal care resource use. Evidence from multiple studies is crucial to identify factors which may be important for predicting length of stay (LOS). The ability to predict LOS is vital for resource planning, decision-making and parent counselling. The objective of this review was to identify which factors are important to consider when predicting LOS in the neonatal unit.

**Design:**

A systematic review was undertaken which searched MEDLINE, EMBASE and Scopus for papers from 1994 to 2016 (May) for research investigating prediction of neonatal LOS. Strict inclusion and exclusion criteria were applied. Quality of each study was discussed, but not used as a reason for exclusion from the review.

**Main outcome measure:**

Prediction of LOS in the neonatal unit.

**Results:**

9 studies were identified which investigated the prediction of neonatal LOS indicating a lack of evidence in the area. Inherent factors, particularly birth weight, sex and gestational age allow for a simple and objective prediction of LOS, which can be calculated on the first day of life. However, other early occurring factors may well also be important and estimates may need revising throughout the baby's stay in hospital.

**Conclusions:**

Predicting LOS is vital to aid the commissioning of services and to help clinicians in their counselling of parents. The lack of evidence in this area indicates a need for larger studies to investigate methods of accurately predicting LOS.

**Strengths and limitations of this study**

- There is little research in the area of predicting length of stay (LOS) and this review investigates the limited evidence for the first time. The same articles were independently identified by two authors.
- This review draws together the limited evidence about predicting LOS and discusses the future work needed.
- A variety of settings, gestational groups and types of analysis were considered in the different studies in this review, and it was not possible to conduct a meta-analysis.

## Background

In the UK, 1 in 10 babies^1^ will require specialist neonatal care. Although the most preterm and smallest babies have the highest risk of mortality, if they survive their length of stay (LOS) in the neonatal unit will be very long. As neonatal survival has improved over recent years, particularly for very preterm babies,^2^ the number of babies requiring long-term neonatal care has increased. Consequently, the workload of the healthcare service, including the total number of days of care required has increased.

The ability to accurately predict LOS in neonatal care is vital for resource planning, commissioning of services and to aid clinicians in their counselling of parents. However, there is a paucity of evidence related to predicting LOS. Much of the limited evidence which does exist is from observational studies which may suffer from bias. Similarly, factors which are identified from a single study or hospital as being important for predicting LOS may be biased by local medical practice within that study or simply be chance findings. Therefore, it is vital that information about the factors which predict LOS is identified from multiple studies to provide robust evidence for future research.

The objective of this review was to identify factors which are important when predicting LOS, and to draw together and discuss the evidence which currently exists.

## Methods

### Selection of studies

MEDLINE, EMBASE and Scopus were searched systematically for papers from 1994 to 2016 (May) which investigated the prediction of mortality and/or LOS. All articles were screened by one author, and a random 10% were screened by a second author to ensure reliability of the reviewing process. Any differences in identified articles were discussed between the two authors. The results presented here relate to the prediction of LOS. The full search strategy is provided in the online supplementary table.

10.1136/bmjopen-2015-010466.supp1 Supplementary table search strategy for the review

### Inclusion criteria

Studies were included which reported risk factors for LOS in the neonatal unit, irrespective of the outcome for the baby, from a multivariable model (eg, logistic regression, linear regression). To be included studies needed to have been undertaken in a human population and have been published in English. Neonatal survival dramatically improved in 1994 with the introduction of routine surfactant use^3^ and antenatal steroids and therefore the search was started from this year. Studies which included data from before and after 1994 were included.

### Exclusion criteria of studies

Exclusion criteria were determined in advance and included:

- Conference proceedings, as these were not peer-reviewed, although efforts were made to investigate if the conference abstract was subsequently published;
- Review articles, letters and editorials as these did not contain original research;
- Countries which were outside the Organisation for Economic Co-operation and Development in 1994 to identify countries with a different demographic profile and healthcare service;^4^
- Clinical trials, as the population would be unlikely to be representative of other babies in neonatal care;
- Wrong study population, for example, investigation of a paediatric or maternal population, or outcome, for example, predicting readmission;
- Specific disease areas (eg, *Escherichia coli* outbreaks or infections) as these babies are very different to other babies in neonatal care;
- Work that was subsequently updated or validation studies.

### Data extraction

A data extraction form was prepared in advance to aid extraction of all necessary information. Information extracted related to: general details of the study (to determine eligibility); study characteristics; study population; outcome; clinical predictors and the quality of the study. Reference lists of included studies were examined for any additional studies which were relevant. Identified prognostic factors were grouped into broad categories of: inherent factors; antenatal treatment and maternal factors; conditions of the baby; treatment of the baby and organisational factors.

### Study quality

The quality of research is known to often be poor in prognostic studies,^5^ and therefore quality was not used as a reason for exclusion from this review. However, study quality was considered and discussed using an adaptation of Quality In Prognostic Studies (QUIPS) tool.^6^ Domains of quality included consideration of: study participation; study attrition; prognostic measurement (eg, measurement, validity, completeness of data); outcome measurement (eg, definition and measurement); risk adjustment and predictors (eg, discussion of missing data) and statistical analysis and reporting (eg, was the model building appropriate, validation considered). A study was considered to be of reasonable quality if potential bias introduced by these domains was minimised as far as practical.

This review was registered with PROSPERO (registration number: CRD42013006020). Ethical approval was not required for this review.

## Results

**Figure 1:**
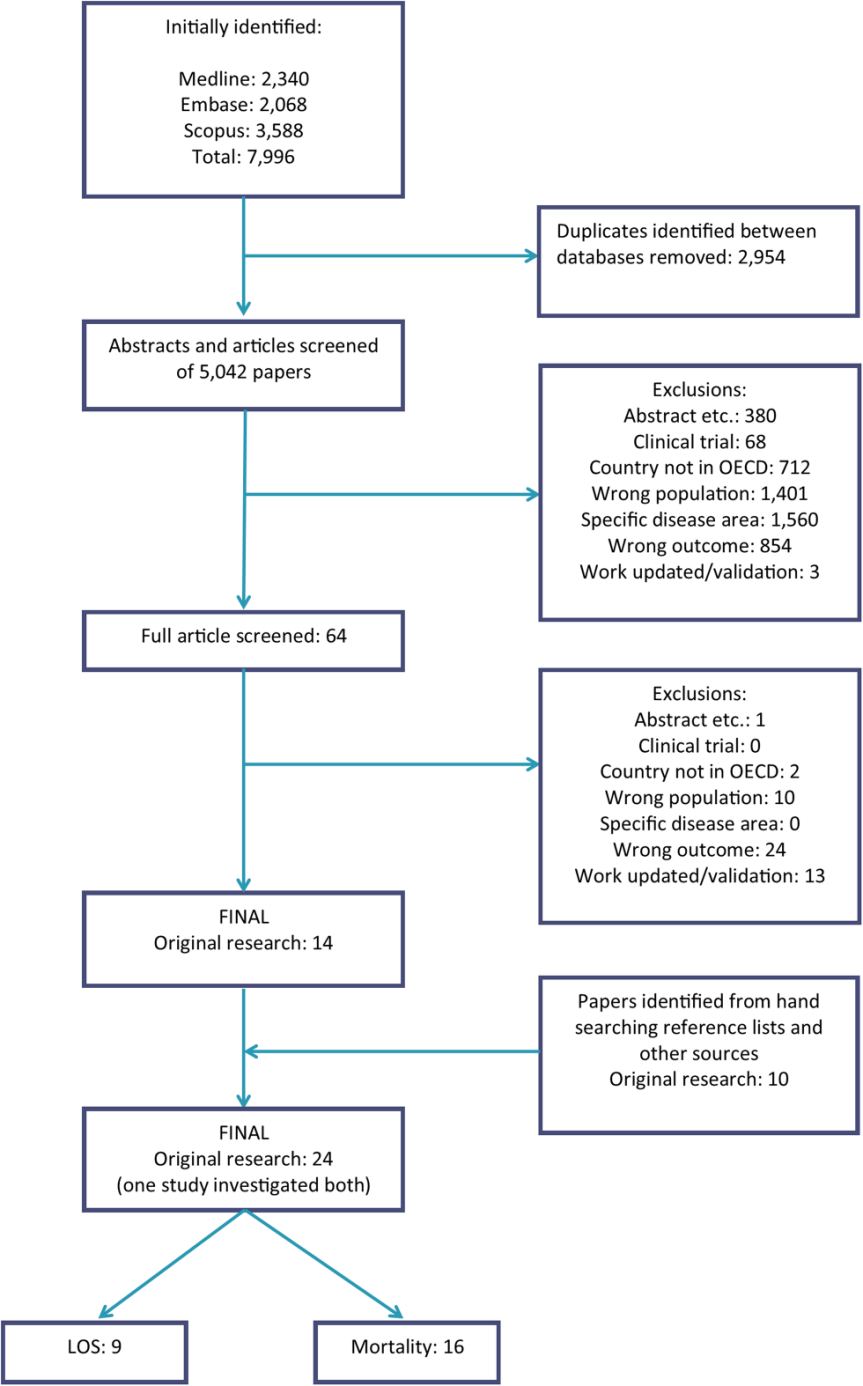
Flow chart documenting the results of the systematic review search. This review focuses on the articles identified which investigated the prediction of LOS. LOS, length of stay; OECD, Organisation for Economic Co-operation and Development.

A total of 7996 studies were identified from a systematic search of MEDLINE, EMBASE and Scopus (see figure 1). After removing duplicates, 5042 studies were screened for inclusion in this review. For 4978 articles it was clear from the title and abstract that they did not satisfy the inclusion criteria. The remaining 64 articles were read in full and manual searching of references, led to a final total of 24 being identified. Of these nine studies investigated the prediction of LOS and are included in this review. Summary characteristics of the studies are provided in table 1.

**Table 1:** Summary characteristics of the nine studies included in this review

	Country of study	Year of publication (data)	Exclusions in study	Number of patients in study	Population investigated	Physical location of study	Model selection	Statistical methods	Model fit methods
Altman *et al*^7^	Sweden	2009 (2004–2005)	Congenital anomalies; death; surgery.	2388	30–34 weeks gestational age	Neonatal units of varying levels of care	Univariate analysis then significant (p<0.2) entered into stepwise	Linear regression	R^2^
Bender *et al*^10^	USA	2013 (1999 and 2002)	Congenital anomalies; death; admitted for comfort care.	293 (validated on 615)	All gestations	Neonatal intensive care unit	Prior knowledge	Accelerated failure time parametric models	Cross validation R^2^
Berry *et al*^14^	Canada	2008 (2002)	Admitted for step down care.	604	All gestations	Neonatal intensive care unit	Prior knowledge	Logistic regression	None, but validation in other centres recommended
Hinchliffe *et al*^15^	UK	2013 (2006–2010)	Ambiguous sex; implausible birth weight.	2723	24–28 weeks gestational age	Neonatal intensive care unit	Prior knowledge	Competing risks	None (acknowledged as weakness)
Hintz *et al*^8^	USA	2010 (2002–2005)	Congenital anomalies; in hospital >1 years; transferred to long-term care.	2254	<27 weeks gestational age	Unclear but likely to be neonatal intensive care due to gestational age	Prior knowledge	Linear mixed model	R^2^
Lee *et al*^9^ (2013)	USA	2013 (2008–2010)	Congenital anomalies; death; surgery.	2012	401–1000 g birth weight	Neonatal intensive care unit	Stepwise selection	Linear mixed model	R^2^
Lee *et al*^11^ (2016)	USA	2016 (2008–2011)	Congenital anomalies; death; surgery; readmitted.	23 551	All babies 401 g–1500 g or 22–29 weeks gestational age plus larger babies meeting specified criteria	Neonatal intensive care units	Prior knowledge then minimum AIC	Negative binomial model with hospital as random effect	Root mean-square error (RMSE)
Manktelow *et al*^12^	UK	2010 (2005–2007)	Death; non-normal care.	4702	23–32 weeks gestational age	Neonatal unit.	Prior knowledge and then change in deviance to decide how to model variables	Quantile regression	Observed vs predicted comparison
Zernikow *et al*^13^	Germany	1999 (1989–1996)	Transfers; deaths.	2144	23–36 weeks gestational age	Unclear but single centre.	Forward stepwise	Artificial neural networks Multiple linear regression	Observed vs predicted comparison

Of the nine identified articles, eight were identified by both authors performing the screening, and the ninth was agreed on after discussion between the authors.

### Description of LOS studies

#### Inclusion and exclusion criteria of LOS studies

Exclusions within the nine studies, included: (major) congenital anomalies (as defined by study authors as no standard exists);^7–11^ deaths in hospital^7^ ^9^ ^11–13^ or before admission to intensive care;^10^ babies who were admitted for comfort care (neither intubation or cardiorespiratory resuscitation was provided);^10^ step down care;^14^ surgery;^7^ ^9^ ^11^ ambiguous sex;^15^ implausible birth weight;^15^ non-normal care pathways;^12^ in hospital >1 year;^8^ previously discharged and readmitted,^11^ transfers,^13^ and transfers to long-term care facilities.^8^

Although most studies excluded infants who died in hospital; two papers included deaths in the calculation of LOS. One paper accounted for this in the methodology implemented^15^ and another acknowledged ‘mortality rates may have introduced bias, since non-survival truncates observed LOS’.^10^ One study which excluded deaths^11^ acknowledged that accounting for deaths in LOS ‘may be particularly complex…’

#### Study populations within LOS studies

Studies investigated a variety of gestational ages and a range of different study settings (table 1) leading to varied populations. Studies appear to have been largely based in intensive care units, although it is difficult to comment on whether individual babies within a study required or received intensive care (eg, mechanical ventilation) as no study stated this explicitly.

#### Prognostic factors in LOS studies

The nine identified studies investigating the prediction of LOS presented a total of 39 prognostic factors. These variables were grouped into broad categories of: inherent factors; antenatal treatment and maternal factors; conditions of the baby; treatment of the baby and organisational factors. Details of the prognostic factors identified by each study are given in table 2.

**Table 2:** Prognostic factors for predicting length of stay included in the analysis of each study

	Altman *et al*^7^	Bender *et al*^10^*	Berry *et al*^14^	Hinchliffe *et al*^15^	Hintz *et al*^8^	Lee *et al*† (2013)^9^	Lee *et al* (2016)^11^	Manktelow *et al*^12^	Zernikow *et al*^13^	Number of studies
Inherent factors										
Birth weight (modelled in multiple ways including categorised, SGA, z score)	X (SGA)	X		X	X	X (+SGA)	X	X	X	8
Congenital anomalies			X				X		X	3
Date/year of birth							X		X	2
Ethnicity/race/nationality						X	X		X	3
Gestational age	X	X		X				X	X	5
Head circumference									X	1
Length of baby at birth									X	1
Multiplicity	X						X			2
Sex		X		X		X	X	X		5
SNAPPE-II‡			X							2
*Any inherent factor*	X	X	X	X	X	X	X	X	X	9
Antenatal treatment and maternal factors										
Antenatal steroids						X	X			2
Diabetes							X			1
Emergency delivery									X	1
Fetal distress						X	X			2
Hypertension						X	X			2
Maternal age	X						X			2
Mode of delivery							X			1
Other maternal/obstetric condition							X			1
Received prenatal care							X			1
*Any antenatal treatment or maternal factor*	X					X	X		X	4
Conditions of the baby										
Admission reason								X		1
Apgar score						X	X			2
Bronchopulmonary Dysplasia					X					1
Hyperbilirubinaemia	X									1
Hypoglycaemia	X									1
Infection	X									1
Respiratory distress syndrome	X									1
Retinopathy of prematurity (stage 3 or higher)					X					1
Sepsis episode or NEC					X					1
Severe morbidity§	X									1
SNAP¶		X								1
SNAPPE-II			X							2
*Any condition of the baby*	X	X	X		X	X	X	X		7
Treatment of the baby										
Surgery while in hospital			X							1
Surgery for patent ductus arteriosus, necrotising enterocolitis, or retinopathy of prematurity					X					1
Umbilical vein catheter									X	1
Ventilation									X	1
*Any treatment of the baby*			X		X				X	3
Organisational factors										
Centre (random effect)					X	X		X		3
Domiciliary care	X									1
Fixed discharge criteria	X									1
Level 3 centre	X									1
Transferred/outborn status			X				X			2
*Any organisational factor*	X		X		X	X	X			5

*The final model is taken to be the SNAP one as this model was validated.

†This study stratified analyses by birth weight, and different variables were used for each stratification. All variables from all models are listed here.

‡The calculation of the SNAPPE-II score includes: MBP; lowest temperature; Po2/FIO2 ratio; lowest serum pH; multiple seizures; urine output; birth weight; SGA and Apgar score. These are a combination of inherent and conditions of baby factors and so SNAPPE II appears in both categories.

§Severe morbidity is defined as: any of: IVH 3-4; ROP>=3; BPD.

¶This is the original SNAP score, devised in 1993, and comprised of 34 items, largely related to the condition of the baby. Examples of items belonging to the score include: heart rate, blood pressure and platelet count.

BPD, bronchopulmonary dysplasia; IVH, intraventricular haemorrhage; MBP, mean blood pressure; ROP, retinopathy of prematurity; SGA, small for gestational age; SNAP, Score for Neonatal Acute Physiology; SNAPPE, Score for Neonatal Acute Physiology Perinatal Extension II.

All nine studies accounted for some form of inherent factor, with the most common being birth weight (88.9%, 8/9), gestational age (55.5%, 5/9) and sex (55.5%, 5/9). Seven studies attempted to account for the condition of the baby. However, there was little consensus on what factor would be appropriate, with variables ranging from those occurring early in the care pathway (eg, admission reason) to those potentially occurring later on (eg, Retinopathy of Prematurity). Similarly, variables such as congenital anomalies were only accounted for by three studies (33.3%, 3/9); however, this often comprised part of the exclusion criteria (55.5%, 5/9).

Organisational factors were considered in 5 (55.5%) studies, with most relating to the setting of the care being received including transfers between units.^14^

#### Study quality of the LOS studies

Quality of research is well acknowledged as an issue in prognostic or prediction studies.^5^ Therefore, an adapted form of the QUIPS tool was used to discuss the quality of the studies (table 3), although poor quality was not used as a reason for exclusion from the review. Domains of bias which were examined included: level of study participation; exclusion and attrition; how the outcome was measured; details about risk adjustment and information about the analyses, specifically if validation was conducted. Study participation was not an issue as all studies used data from routine sources and none actively recruited participants. Attrition caused by infants being transferred out of the area covered by the hospital/study was potentially an issue in all studies except one^10^ which included LOS in other facilities. However, this study^10^ was based in a single centre, and although they lost no infants to attrition, the details about the population they recruited only included care received while within that hospital site.

**Table 3:** Quality assessment of the included studies using a modified version of the QUIPS tool

	Domains of quality				
	Study participation	Study exclusion/attrition	Outcome measurement (eg, definition and measurement)	Risk adjustment and clinical predictors* (eg, missing data)	Statistical analyses and reporting (eg, validation considered)
Altman *et al*^7^	Study is population based (and included 21/34 units in Sweden) but infants were excluded if moved to a hospital not included in study. Data is collected	Infants discharged to other clinics were excluded.	Continuous postmenstrual age at discharge.	Detailed information about how factors were measured.	None mentioned
Bender *et al*^10^	Single centre study.	Transfers were included in the analysis and their LOS in other facilities was included in the total LOS. Sensitivity analyses excluded them.	Continuous LOS (days).	Made use of mortality scores with large number of elements included. Potential issues if there was missing data.	Split sample.
Berry *et al*^14^	Study based in two hospitals. Data extracted from ward registers, charts and patient records.	LOS days after transfer to another centre were not included.	LOS categorised into: <21 days or ≥21 days. No justification for these cut points.	Made use of mortality scores with large number of elements included. Potential issues if there was missing data.	Acknowledgement that future validation required.
Hinchliffe *et al*^15^	Population-based study covering a region of hospitals. Data is extracted from medical records and stored in a routine database used for research purposes.	Minimal losses to follow-up when discharged out of region covered by study. Included in analysis as censored observations.	Continuous LOS (days).	Detailed information about how factors were measured.	Acknowledged that further work is required to assess model.
Hintz *et al*^8^	Population-based study within a large network containing multiple hospitals. Data extracted from a routine database set up for research.	Attrition of infants transferred out of the region covered by study.	Early (lowest quartile of age at discharge) or late discharge (high quartile of age at discharge). No justification for these cut points.	Variables clearly defined. Some factors subjective in measurement (eg, Bells staging for NEC).	Split sample
Lee *et al*^9^ (2013)	Population-based study of a large number of intensive care units.	Attrition from transfers to lower levels of care (acknowledged as causing bias).	Continuous LOS in days (log transformed).	Limited details about variables but most could be measured objectively.	Split sample
Lee *et al*^11^ (2016)	Population-based study in 90% of intensive care units in large American state	Only babies inborn or transferred to unit in study within one day of life.	Continuous LOS (days).	Variables clearly defined and objectively measured. Missing data not discussed.	Split sample
Manktelow *et al*^12^	Population-based study covering a region of hospitals. Data is extracted from medical records and stored in a routine database used for research purposes.	Minimal attrition: when discharged out of region covered by study.	Continuous LOS (days).	Some factors subjectively measured (eg, reason for admission to intensive care).	Acknowledged that future validation needed.
Zernikow *et al*^13^	Single centre study	Transfers excluded from the study.	Continuous LOS (days).	Limited information about variables but most objective to measure.	Split sample

*Unmeasured and unknown confounders are always a potential issue within observational research, so no study has this specifically mentioned.

LOS, length of stay; NEC, necrotising enterocolitis.

Seven studies used continuous LOS/postmenstrual age (PMA) as their outcome.^7^ ^9–13^ ^15^ Two studies categorised LOS, one by dichotomising into <21 days and ≥21 days^14^ and the other by classifying discharge as early or late (lowest and highest quartile of PMA).^8^ The decision of how to model LOS was based on the statistical analysis being implemented. There were no issues in the measuring of LOS, as this is an objective, simple measurement.

Five studies had validated their results by splitting the sample during the initial analysis and holding some data back for validation purposes.^8–11^ ^13^ Two studies acknowledged that further validation was needed before results could be generalised^12^ ^14^ and one acknowledged that further work was needed to assess the modelling techniques.^15^ One study, as part of their analyses, had conducted a preplanned external validation on a model presented in their paper, but concluded that the non-validated model was statistically superior.^10^ Only one study did not mention validation of the results.^7^ Therefore, a strength of these studies was that most addressed the issue of validation in some way.

In general, study quality was considered to be good with low levels of potential bias. There were few issues with study participation as most studies obtained data from medical notes which would introduce a low risk of bias. All studies had a defined outcome which could be objectively measured and so was unlikely to differ between studies indicating no issues of bias. Only one study^7^ did not mention validation of the results, indicating that statistical analyses were well reported. While no formal scoring of study quality has been undertaken here, all studies had a level of quality which indicated there was a low level of bias given the constraints of the study designs.

## Discussion

In recent years, the ability to accurately predict LOS in the neonatal unit has become increasingly important. As neonatal survival has improved, the number of babies requiring long stays in the neonatal unit has increased. However, there has been limited evidence on how to predict LOS, and what factors are important to aid the prediction. This review has provided a systematic search of the literature to consider what factors should be considered in future analyses of LOS.

All of these studies investigated the prediction of LOS, although two studies categorised the outcome,^8^ ^14^ which leads to less informative estimates and therefore using a method which can appropriately model continuous LOS is more useful clinically. It is likely that the choice of how to measure LOS is decided by the selection of statistical method. A variety of methods were used, although surprisingly only one study used a survival analysis approach,^15^ which is often the most popular methodology when measuring time to event.

### Prognostic factors for LOS

All studies accounted for some form of inherent factors which have the advantage of being generally simple and objective to measure, and being present at birth. A prediction for LOS on the first day of life can be made using these factors. However, this prediction may change over time depending on the clinical progress of the baby, and the quality of care provision, during the baby's stay. However, there were a variety of study populations in this review even before adjustment for inherent factors, with predictions for extremely preterm^15^ and for all babies.^10^ ^14^ A prediction model for all babies, such as that proposed by Bender^10^ or Berry^14^ is unlikely to perform well as the babies born near term may have very different reasons for being in the neonatal unit to those born preterm. This was discussed by Lee^11^ who stratified their analyses by different birth weight groups to attempt to group similar babies together. They acknowledged that babies born at a normal birth weight may need further stratification by the reason for their admission, for example: sepsis or respiratory disease.^11^ The approach appears reasonable, and future LOS predictions should focus on groups of babies with similar characteristics, for example, very preterm or very low birth weight, or analyses should be stratified by clinical condition.

It has been acknowledged that while this information from the first day of life is useful,^13^ prediction is generally poor unless perinatal factors^8^ or severity of illness^10^ factors are also considered. However, there was little consensus on what this factor should be, with potential factors ranging from early occurring conditions (eg, reason for admission to intensive care) to those that occurred later in the care pathway (eg, retinopathy of prematurity). Therefore, while it may be important to account for the condition of the baby, there is little agreement over which factors should be used to do so. It is difficult to adjust for conditions which will only be experienced by surviving children. To provide an early prediction of LOS the clinical condition should be an event which occurs early in the care pathway, for example, Apgar score.

Congenital anomalies were not accounted for by many studies, but often formed part of the exclusion criteria within a study, indicating the importance of their consideration. However, there is no accepted list of what constitutes a major anomaly, and the term is often used to refer to a wide and varied range of conditions, making statistical adjustment or exclusions from a study difficult. Some congenital anomalies are unlikely to impact on LOS at all, whereas some severe anomalies or those that require surgery (eg, gastroschisis) may have a significant impact on LOS. Consequently even when studies exclude or adjust for major anomalies it can never be guaranteed that it is a comparison of ‘like with like’. Thus, while congenital anomalies may have an impact on LOS, it is likely too broad a term to include in a LOS prediction model, but it should be considered by clinicians when revising LOS estimates using their clinical judgement.

It is difficult to account for organisational factors, although around half the studies attempted to do this in some way. However, one major issue with organisational factors is the variation between countries. Similarly, even within a country, the level of the unit may not indicate the type of care given to the infant. Despite this, these factors were seen by some authors to be equally or even more important than perinatal risk factors.^7^ This demonstrates the importance of considering the varying levels of care provision within the country of the study. Studies focused in one or two centres such as those by Berry^14^ or Bender^10^ are likely to be inappropriate to draw definitive conclusions from as they may have high levels of loss to follow-up or loss of detail related to the baby's care, causing issues with estimating LOS. Within the UK, neonatal services are focused in clinical networks,^16^ with each network providing the full range of neonatal care. Therefore, it may be appropriate to focus analysis and prediction at a network level to cover all varieties of care, attempting to avoid some of the issues presented by differing organisational factors, and to allow generalisability of the findings. Population-based studies may assist with this; however, these should potentially investigate the use of a random effect term for hospital or equivalent to allow for variation between different healthcare services. Future work should consider the impact of a baby transferring between hospitals on their LOS.

### Thresholds for discharge

Thresholds for determining the timing of discharge informally exist within neonatal medicine. Babies are rarely discharged before they gain the ability to suck and feed (around 35 weeks of gestational age). Irrespective of clinical conditions experienced, most preterm born babies (particularly <32 weeks) are likely to have matured and recovered enough to be discharged at this point, their prematurity being the overwhelming reason for their LOS. For a small number of babies, later occurring conditions (eg, late occurring sepsis, surgical needs) may cause a dramatic increase in their LOS. However, these will not be identifiable for a long period after birth and so potentially, prediction of LOS should be adapted in light of these conditions, if appropriate.

While the LOS of preterm babies is largely determined by their prematurity, normal birthweight babies^11^ and those born closer to term are likely to have varied reasons for their LOS making predictions complex. These babies should be considered separately or adjustment or stratification should be made in any prediction model.

### Clinical use of prediction models

Clinically, prediction models with a smaller number of factors are easier to use,^8^ and this also reflects the concept of statistical parsimony (‘simplicity’). This was seen in the area of predicting neonatal mortality, where complex risk scores, such as the Score for Neonatal Acute Physiology (SNAP), were developed and subsequently simplified to allow easier use.^17^ ^18^ Even following simplification, these risk scores are, at times, still difficult to implement. For example, the simplified SNAP score still requires the assumption that where medical tests are not performed, the results should be considered normal.^17^ Therefore, while accurate prediction is needed, this must be balanced against the need for a simple model, suitable for ‘bedside use’.

Clinical judgement is important and potentially informative for predicting LOS, although this was not possible to investigate here. However, prediction models, such as those identified, are useful because they can provide estimates that are more accurate then clinical judgement and assessment alone.^19^ It is likely that a statistical estimate of LOS, used in conjunction with clinician judgement, for example, when considering congenital anomalies, may provide the best estimate.

### Strengths and limitations of this review

There is little research in the area of predicting LOS and this review investigates the limited evidence for the first time. However, it was difficult to identify a clearly defined population for whom to predict LOS. A variety of settings and gestational groups were considered in the different studies in this review, and it is likely that different gestational ages will require different prediction models, incorporating very different factors. Future research will need to specifically investigate this in large studies.

A meta-analysis of the data presented in this review was not undertaken, due to the varying analyses and adjustments made in each study. Theoretically, an individual patient data meta-analysis could have been undertaken in order to overcome these issues; however, this is known to be difficult, particularly with acquiring the necessary data.^20^ Similarly, it was not possible to investigate publication bias due to the varying analyses and potentially this could have been an important issue. Owing to these limitations, as suggested in other medical areas, a large-scale study may be important and clinically useful.^21^

## Conclusions

The ability to predict LOS would be valuable to parents and families, clinicians and service providers, but it is a complex issue. Inherent factors appear to be the most important to account for, particularly birth weight, gestational age and sex. This information from the first day of life is informative for predicting LOS in a simple model and these estimates are a useful early indicator of LOS.

It may be important to consider revising this initial estimate over time if a late occurring condition dramatically adds to the initial LOS prediction. However, it is hypothesised that many medical conditions will resolve before the point at which the baby is well enough in terms of their prematurity to be discharged. In cases where this assumption is unrealistic more complex (dynamic) risk-prediction models would possibly be required.^22^ Studies predicting LOS should be at a population level to avoid the issue of organisational factors, and to allow generalisability of the findings.
