# Supplementary material for: What factors predict length of stay in a neonatal unit: a systematic review
Source: BMJ Open. 2016 Oct 18;6(10):e010466. doi: 10.1136/bmjopen-2015-010466 (PMC5073598; doi:10.1136/bmjopen-2015-010466)
Supplement: Supplementary table — search strategy for the review [file bmjopen-2015-010466supp_table.pdf]

Supplementary Table: search strategy for the review

Medline		Embase		Scopus
exp Intensive Care Units, Neonatal/ or exp Intensive Care, Neonatal/ or neonatal care.mp	1	"intensive care unit".mp. or exp intensive care/ or exp intensive care unit	1	(TITLE-ABS-KEY(**"intensive care"** OR **"special care"** OR **"NICU"** OR **"high dependency"** OR **"standard care"** OR **"nursery care")**
("intensive care" or "special care" or "NICU" or "high dependency" or "standard care" or "nursery care").mp	2	"neonatal care".mp. or exp newborn care	2	TITLE-ABS-KEY(**"gestational age"** OR **"infant"** OR **"premature"** OR **"preterm"** OR **"baby")**
exp Gestational Age/ or exp Infant, Newborn/ or exp Premature Birth/ or exp Infant, Premature/ or preterm.mp. or baby.mp. or exp Infant, Premature, Diseases	3	("intensive care" or "special care" or "NICU" or "high dependency" or "standard care" or "nursery care").mp	3	TITLE-ABS-KEY**("determinant$"** OR **"risk factor$"** OR **"clinical predictor$"** OR **"predictor$"** OR **"prognostic"** OR **"indicator$"** OR **"prediction"** OR **"probability")**
exp Infant, Low Birth Weight/ or very low birthweight.mp. or exp Infant, Very Low Birth Weight	4	exp prematurity/ or "preterm".mp. or exp gestational age	4	TITLE-ABS-KEY**("length of stay"** OR **"mortality"** OR **"survival"**))
("determinant$" or "risk factor$" or "clinical predictor$" or "predictor$" or "prognostic" or "indicator$" or "prediction" or "probability").mp	5	"low birthweight".mp. or exp low birth weight	5	1 and 2 and 3 and 4
exp Risk Factors	6	"very low birthweight".mp. or exp very low birth weight	6	5 and PUBYEAR > **1993** AND NOT ALL(**animal$** OR **rat** OR **rats** OR **cat** OR **cats** OR **bovine** OR **sheep**)
"length of stay".mp. or exp "Length of Stay"	7	"risk factor".mp. or exp risk factor	7	LIMIT 5 -TO(LANGUAGE, **"English"**)
exp Infant Mortality/ or exp Perinatal Mortality/ or mortality.mp. or exp Hospital Mortality/ or exp Mortality/ or "neonatal mortality".mp	8	("determinant$" or "risk factor$" or " clinical predictor$" or "predictor$" or "prognostic" or "indicator$" or "prediction" or "probability").mp	8	-

exp Survival/ or survival.mp	9	"length of stay".mp. or exp "length of stay"	9	-
1 or 2	10	"infant mortality".mp. or exp infant mortality	10	-
3 or 4	11	"perinatal mortality".mp. or exp perinatal mortality	11	-
5 or 6	12	"hospital mortality".mp. or exp mortality	12	-
7 or 8 or 9	13	"neonatal mortality".mp. or exp newborn mortality	13	-
10 and 11 and 12 and 13	14	1 or 2 or 3	14	-
limit 14 to (english language and humans)	15	4 or 5 or 6	15	-
limit 15 to yr="1994 - Current"	16	7 or 8	16	-
-	17	9 or 10 or 11 or 12 or 13	17	-
-	18	14 and 15 and 16 and 17	18	-
-	19	limit 18 to (human and english language)	19	-
-	20	limit 19 to yr="1994 - Current"	20	-
